# Barriers to Professional Mental Health Help-Seeking Among Chinese Adults: A Systematic Review

**DOI:** 10.3389/fpsyt.2020.00442

**Published:** 2020-05-20

**Authors:** Wei Shi, Zhuozhuo Shen, Siyuan Wang, Brian J. Hall

**Affiliations:** Global and Community Mental Health Research Group, Department of Psychology, Faculty of Social Sciences, University of Macau, Macau, China

**Keywords:** barriers, mental health, help-seeking, Chinese, mental disorder

## Abstract

**Background:**

A large number of Chinese suffer from common mental disorders (e.g., depression, anxiety, stress, and post-traumatic stress disorder), but treatment seeking is typically low in this population. It is unclear what barriers influence professional mental health help-seeking behavior within the Chinese population. Identifying these barriers could assist in implementation science efforts to reach this population.

**Objective:**

This review systematically synthesizes findings related to the barriers to professional mental health help-seeking among Chinese adults.

**Methods:**

Two English language databases (PubMed and PsycINFO) and two Chinese databases (WANFANGDATA and CNKI) were searched to find relevant studies. Quality assessment was conducted in identified studies. Quantitative findings were tabulated and frequently reported barriers were ranked. Primary data obtained from qualitative studies were thematically analyzed.

**Results:**

Of 6,933 citations identified, 17 met inclusion criteria. There were 11 (64%) studies that reported quantitative methods; 3 (18%) employed qualitative research, and 3 (18%) mixed methods. Results indicated that frequently reported barriers to mental health help seeking among Chinese adults included a preference on self-reliance, seeking help from alternative sources, low perceived need toward help-seeking, a lack of affordability, negative attitude toward, or poor experiences with help-seeking. Less frequently mentioned barriers included stigma, families’ opposition, limited knowledge about mental illness, a lack of accessibility, unwillingness to disclose mental illness, and fear of burdening family.

**Conclusions:**

The current review identified a number of key barriers to help-seeking behavior. Effective strategies are needed to promote professional help-seeking among Chinese adults. Additional factors influencing professional mental health help-seeking need to be further investigated, as they may contribute to a better understanding the help-seeking behavior among Chinese.

## Introduction

### A High Prevalence of Mental Disorders in China

There is a high prevalence of mental disorders among Chinese adults. According to a report published in the *Lancet* (1), it was estimated that more than 173 million Chinese adults suffered from a mental disorder, and around 4.3 million were diagnosed as having a severe mental illness in the past year. A psychiatric epidemiological survey conducted by the World Health Organization (WHO) estimated that the 12-month prevalence of mental disorders was 9.1% and 4.3% in Beijing and Shanghai respectively (1). Moreover, in a survey with a sample of 63,004 Chinese adults, the one-month prevalence of mental disorders was 17.5% (2). Based on a sample of 32,552 Chinese adults, a recent national survey indicated that the 12 month and lifetime prevalence of anxiety disorders was 5.0% and 7.6%, respectively (3). Additionally, a survey among 1,158 Chinese older adults in Hong Kong found that there were 11.9% of respondents with common mental disorders in the past month (4). Research conducted among 1,068 Macao Chinese adults found that 8.0% of respondents met criteria for depression during the past two weeks (5), and Hong Kong mental morbidity study with 5,719 Chinese adults indicated that 13.3% of respondents suffered from a common mental disorder in the past week (6). In summary, the burden and prevalence of mental disorders suggests that a large number of Chinese adults may require mental health treatment.

### Reluctance to Help-Seeking Among Chinese Population

Previous studies indicated that many Chinese adults with mental health problems were reluctant to seek professional mental health treatment. For example, based on a report of Hong Kong Health and Welfare Bureau (7), around 95,000 Chinese with mental health problems required professional psychological support, but fewer than 20.0% of them received relevant treatment. According to the result of the Hong Kong Mental Morbidity Survey among 5,719 Chinese adults, the prevalence of common mental disorders was 13.3% and only 26.0% of respondents with common mental disorders (e.g., depression, anxiety, and stress) consulted mental health services in the past year (6). Moreover, previous studies (2) found that around 24.0% of participants were diagnosed with moderate or severe mental disorder among 63,004 Chinese adults, but only 8.0% had ever sought professional help. Additionally, a survey of 1,891 Chinese high school students (age 15–18) in Shantou, Guangdong Province indicated that 7.2% students suffered from severe depressive disorders, but less than 5.0% of students sought informal or formal psychological help (8). Prior studies conducted in Mainland China, Hong Kong, Macao, and Taiwan have consistently demonstrated that few Chinese people sought professional psychological help (9–10).

### Proposed Reasons for Not Seeking Mental Health Help Among Chinese

Previous researchers have explored reasons why Chinese adults were hesitant to seek professional treatment. These mainly follow four aspects: (1) attitudinal, (2) cultural, (3) low perceived need, and (4) structural barriers. More specifically, first, attitudinal barriers included negative attitudes toward mental health help-seeking, having previously poor help-seeking experiences, distrust of mental health professionals and treatment, preference to handle problems themselves, and unwillingness to self-disclose problems (11–14). Second, cultural barriers involve fear of discrimination, losing face concern, self-stigma, public stigma, and choosing alternative treatments (e.g., asking help for physical health, Chinese Traditional Medicine, fortune-tellers, or religious leaders) (8, 15–17). Third, the barrier of low perceived need included trying to handle mental problems by themselves, family’s opposition to help-seeking, under-recognition of the need for treatment, and ignoring the severity of their mental illness (11, 18–20). Finally, structural barriers involved concerns related to insurance, cost, confidentiality, transportation, appointment availability, scheduling, and childcare, insufficient accessibility to psychological health information, and limited resources about available mental health services (15, 21–23).

### Research Gaps

This systematic review aims to fill several research gaps. First, previous reviews of help-seeking barriers concentrated solely on either quantitative studies (24–26) or qualitative studies (27–29). Second, most prior reviews about help-seeking barriers only focused on published studies in English (30–34) and very few of them included related literature in other languages (21). Third, some previous reviews focused on the barriers to help-seeking among Western populations in high income countries, e.g., Canada, United States, Switzerland, and United Kingdom (35, 36). At present, there are limited reviews focused on Asian developing countries or China in particular. More importantly, some studies explored barriers to help-seeking among Chinese (18, 37, 38), but there is no systematic review to synthesize this literature for help-seeking barriers among Chinese. In addition, many researchers suggested barriers to mental help-seeking should be explored in different cultural groups so that implementation of mental health treatments could use this information to efficiently provide more effective mental health services (21, 39–42). This study systematically reviewed barriers to professional mental health help-seeking among Chinese adults reported in the previous literature, in order to provide more extensive insights into help-seeking barriers among Chinese. To our knowledge, this is the first systematic review of barriers toward professional mental health treatment seeking in China.

### Aims and Scope of This Study

The study aims to systematically review previous findings on barriers to professional help-seeking among Chinese adults. Both English and Chinese publications, and qualitative, quantitative, and mixed methods studies were included. In this study, Chinese adults refer to Chinese residents in the greater China region, including mainland China, Hong Kong, Taiwan, and Macao. Barriers are defined as any factors or reasons that prevent people from seeking professional mental health help. Professional mental health help refers to treatment or counseling provided by mental health professionals (e.g., counselors, social workers, psychologists, or psychiatrists). Common mental disorders include depression, anxiety, stress/distress, and post-traumatic stress disorder (PTSD).

## Methods

### Protocol Registration

The study registered at the PROSPERO International Prospective Register of Systematic Reviews (registered ID: CRD42018095475).

### Search Strategy

The following electronic databases were searched for papers published from inception to June 7, 2019: PubMed, PsycINFO, WANFANGDATA, and CNKI. Searching terms aimed to present the primary concepts of “help-seeking,” “mental health,” and “barriers.” Keywords were created for each of these concepts by testing the terminology used in review papers in the help-seeking literature and a thesaurus to find synonyms. Some keywords were also combined with standard MeSH terms from the PubMed and Subject Headings for the PsycINFO database. These search terms were translated from English to Chinese and adapted for Chinese database searches. Moreover, in order to locate more relevant publications, a snowballing method was applied in this review (i.e., checking the reference and citation of identified articles to cover more relevant articles). The detailed searching terms and strategy was displayed in **Appendix 1**: Search terms and Strategies.

### Study Selection and Critical Appraisal

**Figure 1:**
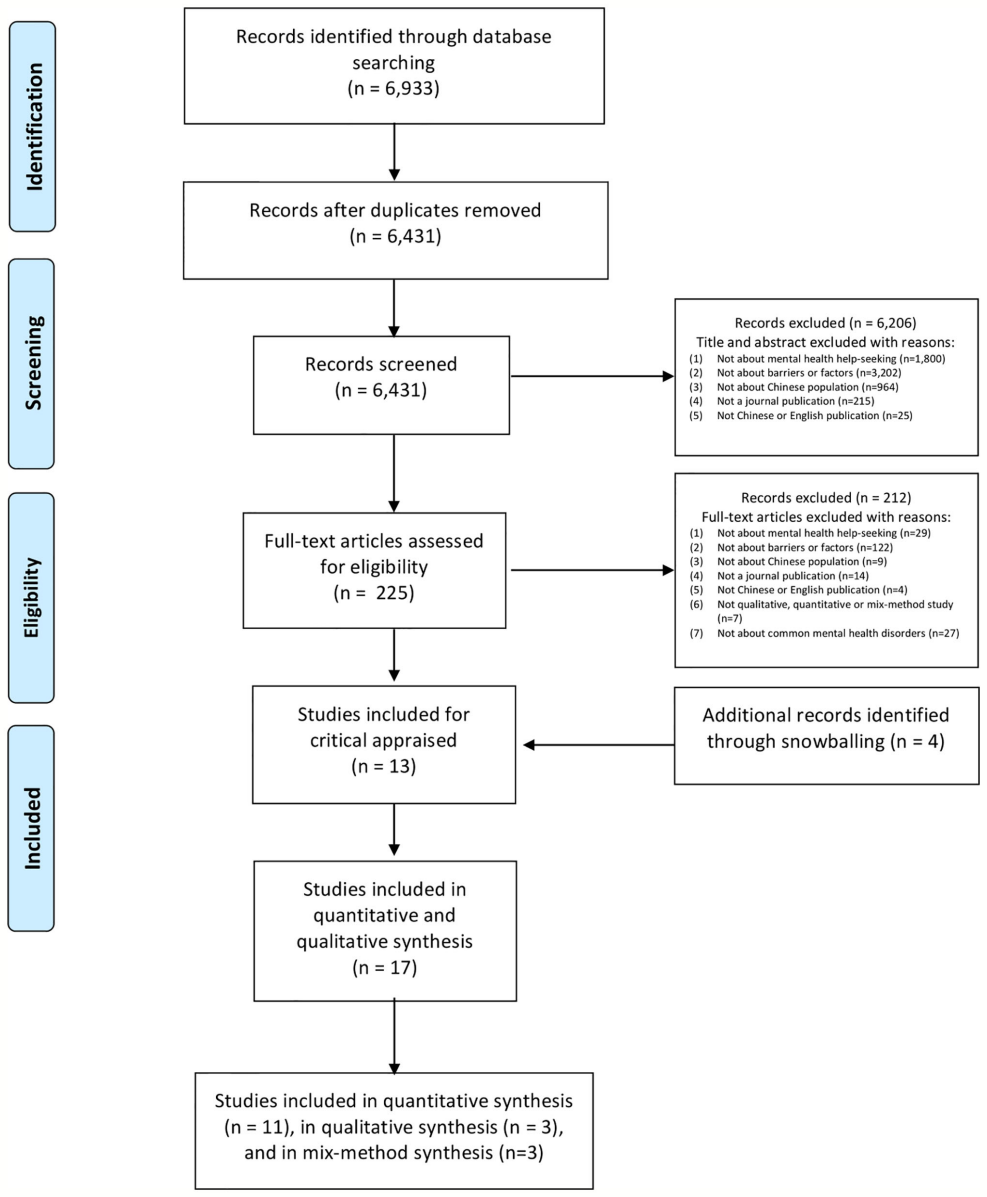
PRISMA flow diagram.

This study followed the PRISMA method (43) to select studies. **Figure 1** displays the PRISMA flow chart for the selection process of the included studies. There were three investigators who screened, evaluate and identified all of the studies together (SW, ZS, and BH). Articles were independently screened by two reviewers (SW and ZS) to evaluate the eligibility. The third investigator (BH) was responsible for cross-checking the final results. Any disagreement on study eligibility was solved *via* discussion.

### Risk of Bias (Quality) Assessment

The Quality Assessment Tool for Observational Cohort and Cross-Sectional Studies (QATOCCSS) (44) and The Critical Appraisal Skills Program (CASP) (45) were applied to assess the quality of 11 quantitative and 3 qualitative studies and the quantitative and qualitative portions of mixed-methods studies respectively. Following the suggestion by Haugen, McCrillis, Smid, and Nijdam (46), QATOCCSS items 7, 8, 10, 12, and 13 were removed because they were not applicable to any identified studies of this review. Using the remaining 9 items, each study was scored as either “good,” “fair,” or “poor” (47). Moreover, the GRADE-CERQual approach was used to assess the methodological limitations, coherence, relevance, and adequacy of qualitative data (48). The certainty of each finding extracted from included studies was then rated as “very low,” “low,” “moderate,” and “high.” There were 2 independent reviewers (SW and ZS) participating in the quality assessment. The third reviewer (BH) cross-checked quality assessment results. Any disagreements were solved *via* discussion until consensus was reached.

### Coding of Studies

Each of the 17 identified studies was coded in a pre-formulated data extraction sheet according to the following characteristics: 1st author name, year published, location (the city/region conducting the study, age of participants (age range or mean), population description (students, adolescents, adults, immigrants, patients), MHS (= mental health status, i.e., what kind of mental disorder the sample of participants have, e.g., depression, anxiety, stress, PTSD), sample size, gender (male, female, both), setting (e.g., high school, community, university, hospital), research method/measure used, specified barriers to help-seeking (description of barrier themes or items as listed in the study).

### Analysis Strategy

The standard method for thematic analysis was applied in participants who endorsed barriers in qualitative literature. Barriers reported by the by quantitative literature were tabulated and the most frequently reported themes extracted (49).

## Results

There were 6,933 published English- and Chinese-language studies that were found in the primary database searches. 6,431 were kept for further screening after removing duplicates. Titles and abstracts were reviewed and 225 potentially relevant studies were retained for full-text review. In total, 13 relevant studies were selected and an additional 4 studies were identified *via* a snowballing method (see **Figure 1**).

### Study Characteristics

**Appendixes 2** and **3** displayed the detailed characteristics of each identified quantitative, qualitative, and mix-method study. The following section briefly introduced the following characteristics including the published year and location of the studies, the research methods applied in the studies, and the participants’ characteristics.

#### Published Year, Location, and Language of Studies

**Figure 2:**
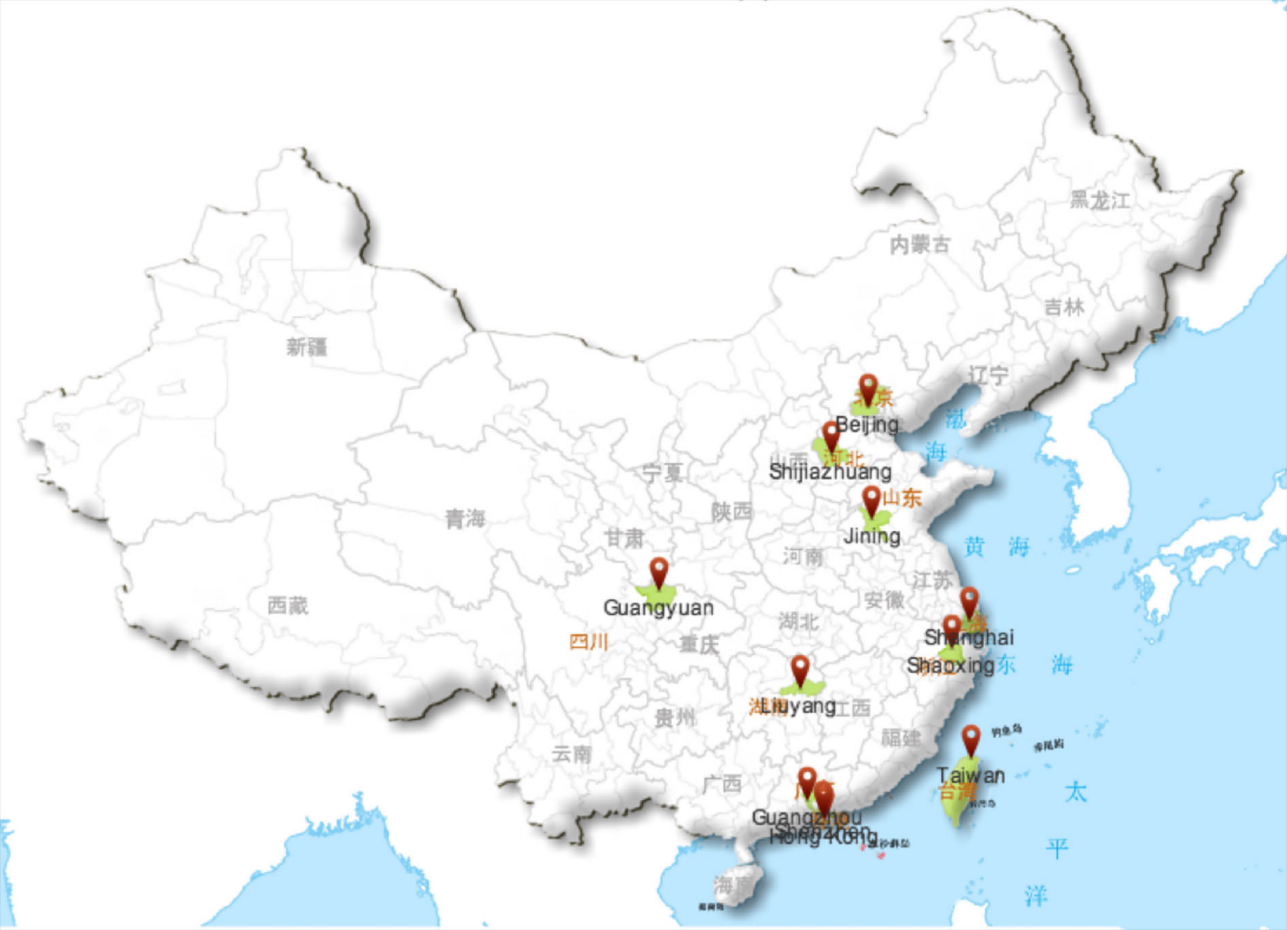
The location of seventeen included studies (N = 11 regions). Red location mark represents the city conducted the study, including **Beijing**; **Shaoxing** of Zhejiang province; **Jining** of Shandong province; **Guangyuan** of Sichuan province; **Shanghai**; **Shenzhen** of Guangdong province; **Liuyang** of Hunan province; **Shijiazhuang** of Hebei province; **Guangzhou** of Guangdong province; **Taiwan**, and **Hong Kong**.

Seventeen identified studies were published between 1998 and 2019. Most of them conducted in Mainland China (n=12) covering 9 cities (i.e., Beijing; Shaoxing of Zhejiang province; Jining of Shandong province; Guangyuan of Sichuan province; Shanghai; Shenzhen of Guangdong province; Liuyang of Hunan province; Shijiazhuang of Hebei province; Guangzhou of Guangdong province). There were 2 studies undertaken in Taiwan and 2 studies conducted in Hong Kong. One study did not report the location of studies, because it collected data *via* a university online forum. **Figure 2** displayed the locations of included studies in the map of China. Most of the included studies were concentrated in coastal cities. Five of the 17 studies were published in Chinese and located in ether the WANFANG or CNKI databases.

#### Research Methods Applied by Studies

Most of the identified studies applied quantitative methods (n=11), the remainder were conducted using qualitative methods (n=3) and mixed methods (n=3).

#### Participants’ Characteristics

*Sample size*: The sample size of identified studies noticeably varied from 16 to 12,333. The qualitative studies (n=3/17) recruited between 16 and 112 participants. The sample sizes of 11 quantitative studies ranged from 100 to 10,179. For 3 mix-method studies, the number of participants was between 9 and 416.

*Participant age*: Participants’ age reported by identified studies varied. The mean age of participants across studies was (19.58–48.65). Some studies just reported the minimum of participants’ age (e.g., ≧15). One study did not report participants’ age.

*Gender*: Most of the studies included both males and females (n=15). There were two studies that only included females.

*Settings and target population*: Seven included studies were conducted in a hospital (n=6) and clinic (n=1). Four studies were based on the university and targeted the student population. Three studies took place in the urban area. Of these, two out of three focused on the adult residents, and one examined both adult residents and migrants. Two studies were conducted in rural areas, of which one examined the adult residents, and another investigated both adolescent and adult residents in rural villages. Finally, there was one study based on households, which targeted adults and children.

*Mental health status of participants*: There were six studies including participants with anxiety and depression. Three studies investigated participants with psychological distress. Another six studies included the sample participants only with depression. One study concentrated on participants with any mental disorder. Only one study did not report the mental health status of the sample.

### Quality Analysis

#### Quality Analysis of Quantitative Studies

A quality assessment was conducted in the eleven quantitative studies and quantitative contents of three mix-method studies *via* the QATOCCSS quality assessment tool (44). Most of them clearly defined the objective, research questions, study population. The participation rate of the eligible person more than 50.0% in almost all of the included studies. Most of the studies included participants from similar populations, the inclusion and exclusion criteria were pre-specified and applied uniformly to all participants. Moreover, exposure and outcome variables were clearly defined, measured, validly, and reliably in most of studies. However, most did not follow high-quality sampling methods. Sample size justification, power description, variance and effect estimates were not reported in most of the studies. Most of the studies did not clearly describe that exposure(s) of interest measured were prior to the outcome(s) being measured. Additionally, key potential confounding variables were measured and adjusted statistically in only few studies. Overall, six studies were considered to be of fair quality with a mean QATOCCSS score of 12.58. Only one study was considered to be of good quality with a QATOCCSS score of 13.5. There were seven studies that were considered to be of poor quality with a mean QATOCCSS score of 8.93. Detailed quality assessment result for the quantitative studies was displayed in **Appendix 5**.

#### Quality Analysis of Qualitative Studies

A quality assessment was also performed on a total of 3 qualitative studies and qualitative contents of 3 mixed-method studies *via* the CASP quality assessment tool (45). All of the included studies clearly described the research type and context. Methods about sampling, data collection and analysis were clearly indicated in most studies. However, most studies did not provide reflexive and sufficient evidence of original interview transcripts. In general, three studies were assessed as having moderate methodological limitations with a mean CASP score of 3.67. Two studies were rated as having no methodological limitations with a mean CASP score of 7. Only one study was considered to have minor methodological limitations with a CASP score of 6. Quality assessments results for qualitative studies were displayed in **Appendix 6**.

### Help-Seeking Barriers in the Quantitative Literature

The twelve included studies used survey methods to collect respondents’ opinion about barriers (i.e., responses to relevant scales, choosing relevant barriers from a list, and ranking the relative importance of barriers).

Barriers reported by included quantitative and mix-methods studies varied across studies. **Table 1** displays the top-rated barriers. The most commonly reported barriers included a preference for self-reliance (11, 50–52), seeking help from alternative resources or treatment (e.g., relying on the family or friend, Traditional Chinese Medicine, physical treatment, and other general practitioners) (11, 50–52), low perceived need toward mental health help (13, 20, 37, 50), distrust in the efficacy of treatment or psychological professionals (18, 56, 57), concerns about the affordability (11, 55). Less frequently reported barriers included no perceived mental health problems (53), negative attitude toward mental health treatment (37), and denying the mental health problems (56).

**Table 1:** Top rated barriers by quantitative and mixed-method studies (n=14).

#	References	Top rated barriers
1	(37)	(1) Low perceived need for treatment (86.5%) (low perceived need) (2) Attitudinal barriers (55.2%) (negative attitude)
2	(18)	(1) Able to resolve distress on one’s own (22.2%) (self-reliance) (2) Distrustful of psychiatrists (17.8%) (distrust)
3	(50)	(1) The problem will go away by itself (53.48%) (low perceived need) (2) I would prefer to handle the problem in another way (34.99%) (using alternative resources)
4	(19)	No quantitative information of barriers provided by this study
5	(11)	(1) I would prefer to handle the problem in another way (52.003%) (using alternative resources) (2) I am concerned about how much money it would cost (23.348%) (affordability)
6	(51)	(1) Seek help from other specialties, e.g. cardiology (39%) (using alternative resources) (2) Used alternative therapies, e.g. traditional Chinese medicine (25.0%) (using alternative resources)
7	(52)	(1) Seek help from friends and family (46.5%) (using alternative resources) (2) Seek help from a general practitioner (20%) (using alternative resources)
8	(53)	(1) No perceived mental distress (23%) (no perceived problems) (2) Prone to solve mental problems by themselves (15%) (self-reliance)
9	(20)	(1) I prefer to deal with issues on my own (56%) (self-reliance) (2) I question how serious my needs are (38.7%) (low perceived need)
10	(54)	No quantitative information of barriers provided by this study
11	(55)	(1) Want to solve it on one’s own (85.2%) (self-reliance) (2) Concern about the cost (43.7%) (affordability)
12	(56)	(1) External attribution for mental illness (i.e. denying mental illness) (denying problems) (2) Worry about the efficacy of mental health treatment and professionals’ ability (distrust)
13	(57)	(1) Solve the problems by themselves (22.3%) (self-reliance) (2) Distrust of psychiatrists (17.8%) (distrust)
14	(13)	(1) Think the problem can be solved by themselves (75.2%) (self-reliance) (2) The problem will go away itself as time goes on (64.49%) (low perceived need)

#1– #11 = Publications in English; #12– #14 = Publications in Chinese; #1, #4, and #10: only quantitative information was assessed in these three mix-method studies.

There were some other barriers endorsed by relatively fewer respondents in quantitative studies, for example, concerns about transportation, appointment, time, and convenience, confidentiality (20, 37), stigmatization, shamefulness, and embarrassment toward mental health treatment (11, 18, 37, 56, 57), worry about discrimination, and loss of face (13, 18), discomfort discussing mental health problems with professionals (50), seeking help from religious organization (52), and family’s opposition toward mental health treatment (56). These findings are displayed in **Appendix 2** in detail.

### Help-Seeking Barriers in the Qualitative Literature

As shown in **Appendix 3**, a total of six studies (i.e., three qualitative studies and three mix-methods studies) identified participants’ perceived barriers to professional mental health help-seeking for mental health problems. A standard thematic analysis was applied in each study by organizing and coding data into 14 themes generated from the terminology used by the review literature (49). The confidence of every theme was assessed by the CERQual assessment tool (48).

The following 14 overarching themes emerged related to barriers to professional mental health help-seeking: (1) seek help/care from other sources; (2) misconceptions of mental illness; (3) self-reliance, not wanting to seek help; (4) low perceived need toward mental health help; (5) perceived low severity of mental illness; (6) fear of stigma to mental illness; (7) negative experiences and attitude toward the treatment; (8) lack of affordability; (9) lack of accessibility; (10) families’ opposition; (11) sociodemographic barriers; (12) unwillingness to disclose mental illness; (13) fear of burdening their families; (14) difficulties in recognizing the mental illness.

**Table 2** summarized CERQual assessment results and key barriers themes emerging from the analysis in order of the frequency of included studies. The most frequently mentioned barrier was seeking help or care from other resources (e.g., Traditional Chinese Medicine doctor, family, and friend), which was reported in four studies (19, 54, 58, 59). Three studies commonly reported barriers relate to misconceptions about mental illness (e.g., physical problem, ideological problem, and bad thinking) (58–60), self-reliance and unwillingness to seek help (19, 37, 54), and low perceived need toward mental health help (19, 37, 54). Two studies reported barriers including perceived low severity of mental illness (37, 58), fear of stigma to mental illness (37, 59), negative experiences and attitude toward the treatment (19, 37), lack of affordability and accessibility (19, 37), families’ opposition to treatment-seeking (37, 58), and sociodemographic barriers (e.g., older age, lower education background, and masculinity) (37, 58). Finally, only one study cited issues related to unwillingness to disclose mental illness, fear of burdening their families (59), and difficulties in recognizing the mental illness (54). In addition, according to the CERQual assessment of confidence in the evidence, the majority of extracted key barriers themes (n=12) were considered to have moderate confidence. The rest of the barrier themes (n=2) were evaluated to have low confidence. **Appendix 4** displays detailed barrier themes and CERQual assessment results in detail.

**Table 2:** Key barriers themes and CERQual assessment among qualitative (n=3) and mix-method studies (n=3).

#	Barrier theme	References	CERQual Assessment of Confidence in the Evidence
1	Seeking help/care from other resources	(19, 54, 58, 59)	Moderate confidence
2	Misconceptions for mental illness	(58–60)	Moderate confidence
3	Self reliance and unwillingness to seek help	(19, 37, 54)	Moderate confidence
4	Low perceived need for mental health help	(19, 37, 54)	Moderate confidence
5	Perceived low severity of mental illness	(37, 58)	Moderate confidence
6	Fear of stigma to mental illness	(37, 59)	Moderate confidence
7	Negative experiences and attitude toward the treatment	(19, 37)	Moderate confidence
8	Lack of affordability	(19, 37)	Moderate confidence
9	Lack of accessibility	(19, 37)	Moderate confidence
10	Families’ opposition	(37, 58)	Low confidence
11	Sociodemographic barriers	(37, 58)	Low confidence
12	Unwillingness to disclose mental illness	(59)	Moderate confidence
13	Fear of burdening their families	(59)	Moderate confidence
14	Difficulties in recognizing the mental illness	(54)	Moderate confidence

For three mixed-method studies (19, 37, 54), in which only quantitative information was analyzed.

This systematic review followed the PRISMA statement (43). The PRISMA checklist was displayed in **Appendix 7**.

## Discussion

This review identified a variety of barriers to professional mental health help-seeking among Chinese adults. However, the results indicated a scarcity of high-quality research in this area. Most of the research concentrated on quantitative rather than qualitative methods to collect data. In the following sections, the most notable barriers to professional mental health help-seeking from the present systematic review will be discussed.

### A Preference of Self-Reliance

In the present study, both qualitative and quantitative data indicated that Chinese people preferred to rely on themselves to deal with mental health problems, rather than to seek professional mental health help or treatment. This finding was consistent with several previous studies about mental health help-seeking among Chinese (15, 61, 62). Moreover, there was a prior study indicating that Chinese mental patients were afraid of disclosing their mental health problems to others, which enhanced a preference of self-reliance for solving mental health problems (38). Another cross-sectional study in Hong Kong found that a preference for self-reliance during difficult times might result in the deterioration of mental problems and worsening outcomes, including suicide or hurting others (63).

### Seeking Help From Alternative Sources

In this study, seeking help from alternative sources emerged in both the qualitative and quantitative studies as one of the most noticeable barriers to professional mental health help-seeking among Chinese. Some previous reviews about help-seeking barriers among the general population have not included this finding (30, 49, 64). There were two studies focusing on help-seeking barriers among Chinese inpatients, which consistently mentioned that some Chinese preferred to seek help from Traditional Chinese Medicine doctors or clinics rather than mental health professionals when they suffered from mental health problems (58, 59). Two studies conducted with Chinese adolescents and adults indicated that seeking help from families or friends was always the first option to handle mental health problems (19, 54). This finding is in line with a previous survey about help-seeking behavior among Chinese psychiatric patients attending a public psychiatric outpatient clinic or private psychiatric clinic that found Chinese would seek mental health treatment from Traditional Chinese Medicine (e.g., Chinese herbalists and acupuncturists) and folk religion (e.g., temples and fortune tellers) (65). While we conceptualized this as a barrier to professional mental health treatment, we also acknowledged the cultural underpinnings and legitimacy of this help-seeking practice.

### Low Perceived Need for Professional Mental Health Help

The present review found that low perceived need toward professional help was identified as one of the prominent barriers in qualitative and quantitative studies. In qualitative research about help-seeking barriers among urban Chinese, respondents reported that “psychological counseling or mental health services are not necessary and essential,” which provided support for this finding (19). This finding has been reinforced by previous studies in mainland China (66) and Taiwan (67), which indicated that professional mental health help was not necessary or essential because some Chinese thought their mental health problems were not critically important, and tried to normalized/deny their mental health issues. Additionally, this finding coincided with a prior review about Chinese perspective on primary care, which mentioned that some Chinese under-recognized the severity of their mental health problems and the need for professional mental health treatment (21).

### A Lack of Affordability for Professional Mental Health Help

A lack of affordability for professional mental health help or treatment has been widely discussed in the previous literature. For example, one qualitative research study in Beijing pointed to the lack of affordability as a key barrier to mental health help-seeking among Chinese, in which some respondents said: “The first thing I thought about mental health service was the cost. It must be very expensive, more expensive than seeking general doctors. We cannot afford it at all.” (19). Worrying about the cost of mental health treatment has also identified previously in the context of help-seeking in another two cross-sectional studies (68, 69). In addition, another semi-structured qualitative interview conducted among Chinese psychiatrists and patients also supported this finding, which indicated that concerns about high treatment fee was one of the obstacles to seek professional mental health help (59).

### Negative Attitude or Experience About Professional Mental Health Help

Negative attitude or experience about professional mental health help was another typical barrier particularly in the studies of urban residential population, this finding was consistent with the previous study among adults in the urban area (37). Based on the urban areas, some studies reported that negative attitude (e.g., doubting the efficacy of treatment, distrust the psychological professional, no confidence in mental health help, or trying to ignore mental illness) prevented patients from further seeking relevant mental treatment (52, 56, 70). Additionally, previous research among Chinese adults with mental disorders suggested that poor mental health treatment experiences (e.g., inefficient treatment, bad counseling setting, non-professional counselor, misdiagnosis by doctors) resulted in non-usage or fear of other mental health help/treatment (71).

## Limitations

Four limitations in the current study could be considered in future research. First, this study applied frequency of themes and the number of studies reporting each theme in qualitative research, and the top-rated barriers in quantitative research. The frequencies might reveal the relative importance of themes/topics but this may not necessarily be the case. Frequency counts of qualitative research may overemphasize the significance of topics that were reported in various different forms (e.g., a preference of self-reliance). However, the benefit of this method provided a valuable starting point for producing future research and specifically for indicating potentially suitable aims for interventions to promote mental health help-seeking (49). Second, the review was imitated to only reported barriers to help-seeking among permanent Chinese residents in greater China. Results may not generalize to other Chinese populations, e.g., overseas Chinese, American-born Chinese (12). Third, as a large proportion of the identified quantitative studies in this review were graded as poor or fair, the findings related to this review should be read with caution. Future studies should employ more methodologically rigorous research designs and reporting to improve the evidence of barriers to mental health help-seeking in the Chinese population. Finally, the 17 papers identified only covered help-seeking barriers among Chinese adults with common mental disorders, such as depression, anxiety, and mood disorders. Future studies are recommended that explore help-seeking barriers for other mental disorders, (e.g., psychotic disorders and autism, obsessive-compulsive disorder) which would help to better understand help-seeking among Chinese adults.

## Conclusions

This study summarized key barriers to professional mental health help-seeking among Chinese adults. These included a preference of self-reliance, seeking help from alternative sources, low perceived need for help-seeking, a lack of affordability, negative attitudes or past experiences with mental health help-seeking. These were prominent and noticeable barriers reported in qualitative, quantitative and mixed-method studies. Included studies also mentioned other barriers that might play a relatively minor role in influencing help-seeking, including stigma (e.g., self-stigma and social stigma), families’ opposition, limited knowledge of mental illness, a lack of accessibility (e.g., concerns about time, appointment, transportation, inconvenient location of relevant organization), unwillingness to disclose mental illness, and a fear of burdening their families.

The findings suggested several ways forward. First, in respect to strategies for enhancing help-seeking among Chinese and addressing a preference of self-reliance, one of the most efficient approaches was to provide a public mental health program to increase mental health literacy and normalizing seeking care from mental health professionals when indicated (72). A second approach could involve enhancing the positive publicity and advertisement of professional mental health help-seeking, which could reduce concerns about affordability and accessibility about professional mental health help (73). The final potential approach might be to increase the mental health workforce and to provide relevant professional training programs for cultivating more relevant service providers in these areas (5, 21).

However, based on different aspects of the help-seeking process, barriers might vary; therefore, further investigation is needed about the operation of those barriers in different levels of help-seeking process (49). A more sophisticated investigation of other barriers to help-seeking is required to advance the field, which could be beneficial for developing a more efficient and effective mental health treatment. For example, a qualitative study mentioned the fear of burdening their families could be an important barrier to help-seeking among Chinese (59), which was not yet measured in any quantitative studies. Recent research found that substantial increases in mental health provision did not decrease the prevalence of mental disorders among the population of four developed countries (74). Future studies should investigate whether increased help-seeking provides benefit to the Chinese population. Culture must be considered when identifying signs and symptoms of distress (75) and in the design and conduct of appropriate treatments to ameliorate these symptoms when indicated. In addition, more research about help-seeking barriers among Chinese is needed within non-coastal cities and in rural areas of China, where barriers to help-seeking remain unclear. The majority of studies were conducted in eastern coastal metropolises (54).

## Author Contributions

All authors made substantial contributions to this study. BH and SW contributed to the conception and design of review. BH, SW, and ZS equally contributed to the literature analysis, studies assessment result interpretation, and drafting manuscript. SW, ZS, and WS contributed to general literature searching and organization. BH and SW contributed to manuscript revision. All authors read and approved the submitted version.

## Funding

This paper is supported by the University of Macau and Macao (SAR) government through the grant MYRG2019-00120-FSS (PI: Hall). The funders played no role in the conduct of the study.

## Conflict of Interest

The authors declare that the research was conducted in the absence of any commercial or financial relationships that could be construed as a potential conflict of interest.
