# Supplementary material for: Barriers to Professional Mental Health Help-Seeking Among Chinese Adults: A Systematic Review
Source: Front Psychiatry. 2020 May 20;11:442. doi: 10.3389/fpsyt.2020.00442 (PMC7251144; doi:10.3389/fpsyt.2020.00442)
Supplement: Supplementary file 1 [file DataSheet_1.docx]

**Appendix 1: Search terms and Strategies**

***SEARCH 1:*** ***PsycINFO (conducted on March 6, 2018)***

1. Barrier$ OR Promot$ OR Obst$ OR Support$ OR Cause$ OR Treatment Barrier$ OR Factor$ OR Discrimin$ OR Difficult$ OR Stigma$ (Subject Heading)

**AND**

2. Mental Health OR Mental Disorders (Subject Heading)

**AND**

3. Helpseek$ OR Seek$ help OR Seek$ OR Treatment Seek$ OR Help Seeking Behavior OR Help$

**AND**

4. Chin$ OR Hong Kong OR Maca$ OR Mainland Chin$ OR Taiwan$

***SEARCH 2: PubMed (conducted on March 6, 2018)***

1. Barrier* OR Promot* OR Obst* OR Support* OR Cause* OR “Treatment Barrier*” OR Factor* OR Discrimin* OR Difficult* OR Stigma* [MeSH]

**AND**

2. “Mental Health” OR “Mental Disorders” [MeSH]

**AND**

3. Helpseek* OR “Seek* help” OR Seek* OR “Treatment Seek*” OR “Help Seeking Behavior” OR Help*

**AND**

4. Chin* OR “Hong Kong” OR Maca* OR “Mainland Chin*” OR Taiwan*

***SEARCH 3: CNKI (******中國知網)*** ***(conducted on March 7, 2018)***

1. KY = '心理' OR KY = '心理健康' OR KY = '精神' OR KY = '精神健康' OR KY='精神卫生' OR KY = '焦虑症' OR KY = '焦虑' OR KY = '抑郁症' OR KY = '抑郁' OR KY = '压力' OR KY = '情绪' OR KY = '情绪障碍' OR KY = '心理障碍' OR KY = '污名' OR KY = '羞恥'

**AND**

2. KY = '求助' OR KY = '求助意愿' OR KY = '求助行为' OR KY = '帮助' OR KY = '咨询' OR KY = '咨商'

**AND**

3. KY='障碍' OR KY='治疗阻碍' OR KY='困难' OR KY='因素' OR KY='原因' OR KY=污名' OR KY='偏见' OR KY='歧视'

***Translation for the SEARCH 3: CNKI (中國知網) above***

1. KY='Mental' OR KY=Psychological Health' OR KY = 'Mental' OR KY = 'Mental health' OR KY='Mental hygiene' OR KY='Anxiety symptom' OR KY='Anxiety' OR KY='Depressive symptom' OR KY='Depression' OR KY='Stress' OR KY= 'Emotional disorder' OR KY='Emotional problems' OR KY= 'Psychological disorder ' OR KY = 'Stigma' OR KY = 'Shame'

**AND**

2. KY = 'Help-seeking' OR KY = 'Help-seeking intention' OR KY = 'Help-seeking behavior' OR KY = 'Help' OR KY = 'Counseling' OR KY = 'Psychological treatment'

**AND**

2. KY='Barriers' OR KY='Treatment Obstacles ' OR KY='Difficulties' OR KY='Factors' OR KY='Reasons' OR KY='Stigma' OR KY='Bias' OR KY='Discrimination'

***SEARCH 4: WANFANGDATA (******萬方數據) (conducted on March 7, 2018)***

(关键词:(心理 or 心理健康 or 精神 or 精神健康 or 精神卫生 or 焦虑症 or 焦虑 or 抑郁症 or 抑郁 or 压力 or 情绪 or 情绪障碍 or 心理障碍 or 污名 or 羞恥 )*关键词:(求助 or 求助意愿 or 求助行为 or 帮助 or 咨询 or 咨商)* 关键词: (障碍 or 治疗阻碍 or 困难 or 因素 or 原因 or 污名 or 偏见 or 歧视))*Date:-2018

***Translation for SEARCH 4: WANFANGDATA (萬方數據) above***

(Keyword:(Mental or Psychological Health or Mental or Mental health or Mental hygiene or Anxiety symptom or Anxiety or Depressive symptom or Depression or Stress or Emotional disorder or Emotional problems or Psychological disorder or Stigma or Shame)*Keyword:(Help-seeking or Help-seeking intention or Help-seeking behavior or Help or Counseling or Psychological treatment)* Keyword: (Barriers or Treatment Obstacles or Difficulties or Factors or Reasons or Stigma or Bias or Discrimination))*Date:-2018

***SEARCH 5: The Snowballing Methods (conducted on March 21, 2018)***

(1). Checking the references of identified articles to locate more articles that were not identified via the previous searching;

**AND**

(2). Checking authors have cited the identified articles via the Web of Science.
