# Supplementary material for: Barriers to Professional Mental Health Help-Seeking Among Chinese Adults: A Systematic Review
Source: Front Psychiatry. 2020 May 20;11:442. doi: 10.3389/fpsyt.2020.00442 (PMC7251144; doi:10.3389/fpsyt.2020.00442)
Supplement: Supplementary file 2 [file DataSheet_2.doc]

**Appendix 2: Quantitative studies included in the review**

**Appendix Table 2: Included quantitative studies on barriers (n=14)**

| **#** | **Authors** | **Year** | **Loc** | **Age** | **Population** | **MHS** | **N** | **Sex** | **Setting** | **Measure used** | **Barriers listed by study** |
| --- | --- | --- | --- | --- | --- | --- | --- | --- | --- | --- | --- |
| 1 | Andrade et al. | 2014 | BJ  SH  & SZ | 18-88 | Adults | Anxiety  & Depression | 12,333 | M/F | Urban area | Participants who reported no use of mental health services were asked whether there was a time in the past 12 months when they felt they might have needed to see a professional for problems with their emotions, nerves or mental health. Those with ‘perceived need’ were then asked about structural and attitudinal barriers. | Percentage of participants reporting barriers (%) by barrier type (~% estimates from appendix table A1 and table A2 provided):  Barriers to use mental health treatment  1.1 Low perceived need for treatment (N1=86.5%; N2=93.1%, N3= 56.4%; N4=44.7%)   1. The problem went away by itself, and I did not really need help.   1.2 Any structural barriers (N1=9.7%; N2=2.7%; N4=0.4%)   1. My health insurance would not cover this type of treatment. 2. I was concerned about how much money it would cost. 3. I was unsure about where to go or who to see. 4. I thought it would take too much time or be inconvenient. 5. I could not get an appointment. 6. I had problems with things like transportation, childcare, or scheduling that would have made it hard to get to treatment   1.3 Any attitudinal barriers (N1=8.7%; N2=6.1%; N3=43.6 %; N4=55.2%)   1. I thought the problem would get better by itself 2. I didn’t think treatment would work. 3. I was concerned about what others might think if they found out I was in treatment. 4. I wanted to handle the problem on my own. 5. I was scared about being put into a hospital against my will. 6. I was not satisfied with available services. 7. I received treatment before and it did not work. 8. The problem didn’t bother me very much.   *Notes:*   1. N1=Participants came from Beijing or Shanghai with serious mental disorders (sample = 211 respondents) 2. N2=Participants came from Beijing or Shanghai with moderate or mild mental disorders (sample = 211 respondents) 3. N3=Participants came from Shenzhen with serious mental disorders (sample = 593 respondents) 4. N4=Participants came from Shenzhen with moderate or mild mental disorders (sample = 593 respondents) |
| 2 | Boey | 1999 | BJ & SH | 18-24 | Students | Anxiety & depressive mood. | 494 | M/F | University | Participants were asked if they would seek mental health help when they suffered from anxiety and depressive mood. | Percentage of participants endorsing barrier (reasons for not seeking psychiatric consultation) (%).   1. Able to resolve distress on one's own (22.2%) 2. Distrustful of psychiatrists (17.8%) 3. Problem not serious enough (16.7%) 4. Other alternatives available (11.4%) 5. Stigmatization, face-losing, shameful (9.6%) 6. Consultation itself stressful (8.5%) 7. Setting terrifying (6.1%) 8. Not willing to self-disclose (4.0%) 9. Others (including avoiding seeing psychiatric patients, not being used to the clinical setting, not knowing what psychiatric consultation was, troublesome procedure, inconvenient location, etc.) (3.7%) |
| 3 | Chen | 2012 | BJ | 18-70 | Permanent residents and  migrants | Psychological distress | 1,474 | F | Urban area | Participants were asked the perceived barriers to seeking professional help (e.g." what barriers do people perceive when they attempt to access professional mental health services?" & "Is there a strong resistance to seeking help through professional means? ") | Percentage of perceived barriers to seeking professional help among the whole sample (N=1,440) & subsamples with distress (N=56)  1. Accessibility (5.01% & 21.53%)   1. I am unsure about where to go or who to see. (3.98% & 15.17%) 2. There is no professional service provider within my area (1.59% & 7.03%)   2. Affordability (16.95% & 27.18%)   1. I am concerned about how much money it would cost. (10.41% & 23.26%) 2. I think it would take too much time. (8.23% & 13.57%)   3. Refusal to recognize need (63.60% & 65.80%)   1. The problem will go away by itself. (53.48% & 56.81%) 2. I would prefer to handle the problem in another way. (34.99% & 35.71%)   4. Lack of trust (4.98% & 22.04%)   1. I do not think professional treatment would help. (3.80% & 16.16%) 2. I fear being hospitalized against my will. (1.40% & 5.88%)   5. Embarrassment or stigma (4.52 % & 15.75%)   1. I am concerned about what people would think if they found out I was in treatment. 2. (2.86% & 9.8%) 3. I do not feel comfortable discussing my problems with a professional. (1.82% & 6.62%) |
| 4 | Chen | 2018 | BJ | 18-90 | Adult residents | Psychological distress | 2,558 | M/F | Urban area | No quantitative information of barriers provided by this study | No quantitative information of barriers provided by this study |
| 5 | Chen, Xu, & Wu | 2019 | HK | M=46.425 | Adults & Children | Psychological distress | 4,589 | M/F | Household-based | Participants were asked the perceived barriers to seeking professional help via the following hypothetical question: “Here are some reasons that people give for not seeking professional help for emotional problems or mental distress even when they might need it. Do any of these statements apply to you?”  The statements address two perceived structural barriers: accessibility (“I am unsure about where to go or who to see”, “There is no professional service provider within my area”) and affordability (“I am concerned about how much money it would cost”, “I think it would take too much time”). They also address three cultural barriers: lack of perceived need (“The problem will go away by itself”, “I would prefer to handle the problem in another way”), lack of trust (“I do not think professional treatment would help”, “I fear being hospitalized against my will”), and embarrassment or stigma (“I am concerned about what people would think if they found out I was in treatment”, “I do not feel comfortable discussing my problems with a professional”). | Percentage of perceived barriers to seeking professional help (%)  1. Structural barriers  1.1 Accessibility (18.041%)   1. I am unsure about where to go or who to see (13.331%) 2. There is no professional service provider within my area (6.946%)   1.2. Affordability (33.967%)   1. I am concerned about how much money it would cost (23.348%) 2. I think it would take too much time (21.179%)   2. Cultural barriers  2.1 Lack of perceived need (59.319%)   1. The problem will go away by itself (47.495%) 2. I would prefer to handle the problem in another way (52.003%)   2.2 Lack of trust (18.305%)   1. I do not think professional treatment would help (14.211%) 2. I fear being hospitalized against my will (6.240%)   2.3 Embarrassment or stigma (14.288%)   1. I am concerned about what people would think if they found out I was in treatment (10.653%) 2. I do not feel comfortable discussing my problems with a professional (6.263%) |
| 6 | Chen et al. | 2013 | TW | ≧ 18 | Outpatient | Depression & anxiety | 100 | M/F | Hospital | 1. Alternative therapies  There were 33.3% of patients who delayed their seeking psychiatric care because of seeking the alternative therapies. These patients were asked to report their alternative therapies.  2. Seeking help from other specialties  There were 76% of patients who sought help from other specialties instead of professional psychiatrist. These patients were asked to report the type of visited other specialties. | Percentage of participants endorsing the alternative therapies and other specialities (%).  1. Type of used alternative therapies (33%)   1. Traditional Chinese medicine (25.0%) 2. Massage therapy (7.0%) 3. Tui nan therapy (6.0%) 4. Vitamin therapy (5.0%) 5. Individual psychotherapy (5.0%) 6. Spiritual healing (4.0%) 7. Aromatherapy (4.0%)   2. Type of seeking help from other specialties (76.0%)   1. Cardiology (39.0%) 2. Gastroenterology (18.0%) 3. Neurology (18.0%) 4. Emergency department (9.0%) 5. Family practice (4.0%) 6. Endocrinology and metabolic disorders (3.0%) 7. Otolaryngology (1.0%) |
| 7 | Chin et al. | 2015 | HK | ≧ 18 | Adults | Depressive symptom | 10,179 | M/F | Clinics | Participants were asked their help-seeking preferences if they had depression (participants were permitted to choose more than one option.) | Percentage of overall participants (N = 10,179) & participants with depressive symptom (N = 1,079) reporting help-seeking preferences (%)   1. Friends and family (46.5% & 41.1%) 2. Religious organization (8.4% & 8.3%) 3. Social worker (10.1% & 10.8%) 4. General practitioner (19.9% & 20.0%) 5. Community service (3.1% & 3.8%) 6. TCM: Traditional Chinese medicine practitioner (3.9% & 4.3%) 7. Telephone hotline (2.3% & 2.7%) 8. Psychiatrist (24.9% & 25.7%) 9. Psychologist (22.8% & 24.2%) 10. Others (0.8% & 0.6%) |
| 8 | Han et al. | 2015 | TW | Mean = 62.2 | Patients with various physical illnesses | Depression. | 230 | M/F | Hospital | Participants would conceal their mental distress and did not seek mental health help. These participants were asked the reasons why they conceal their mental distress. | Percentage of overall participants (N = 109) & depressed participants (N = 35) reporting the reasons for non-disclosure of mental distress by depressive condition (%)  1. Medical-related (44% & 57%)   1. Medical visits are too short (14% & 14%) 2. Doctors cannot solve my problems (13% & 22%) 3. Doctors do not pay attention to psychological issues (N=17% & 22%)   2. Personal-perceived (50% & 38%)   1. No perceived mental distress (23% & 7%) 2. Prone to solve mental problems by themselves (15% & 17%) 3. Other reasons (i.e. not familiar with the doctors, feeling shameful if telling their distress, not necessary, not used to disclose to the doctors, doctors do not want to listen). (12% & 14%)   3. Sociocultural related (8, 6% & 5%)   1. Disclose to family or friends instead (4% & 2%) 2. Feeling shameful (2% & 2%) |
| 9 | Han, Batterham, Calear, & Ma | 2018 | / | M=19.58 | Students | Depression, & Anxiety | 208 | M/F | University online forum | Participants were asked the reasons for not seeking professional help: Participants who endorsed (0) “Highly unlikely” or (1) “Unlikely” on intentions to seek help from mental health workers or psychiatrists were further asked about their reasons for not seeking help with the question: “Why would you be unlikely to seek help from a psychiatrist (mental health worker)?” . The following choices were provided: “I prefer to deal with issues on my own” ; “I question how serious my needs are” ; “I worry about what others will think of me” ; “I have concerns about privacy” ; “I get a lot of support from others, such as family and friends” ; “I don't know how to access a psychiatrist” ; “I don't think a psychiatrist could help me” ; “I don't have access to a psychiatrist where I live” ; “It would cost too much money” ; “It would take too much time or effort” ; “I would not want to be given medication” ; “I would be afraid of being admitted to a mental institution” ; and “Other”, which enabled participants to enter other reasons not included on the list. | Barriers to seeking help from mental health professionals (from mental health workers & psychiatrists)   1. I prefer to deal with issues on my own (56% & 48%). 2. I question how serious my needs are (38.7% & 54.7%). 3. I worry about what others will think of me (21.3% & 25.3%). 4. I have concerns about privacy (28% & 36%). 5. I get a lot of support from others, such as family and friends (48% & 50.7%). 6. I don't know how to access a psychiatrist / psychologist (24% & 24.0%). 7. I don't think a psychiatrist/mental health worker could help me (28% & 33.3%). 8. I don't have access to a psychiatrist/ mental health worker where I live (20% & 20.0%). 9. It would cost too much money (22.7% & 22.7%). 10. It would take too much time or effort (28% & 24%). 11. I would not want to be given medication (6.7% & 9.3%). 12. I would be afraid of being admitted to a mental institution (12% & 29.3%). |
| 10 | Qiu et al. | 2018 | GY | >16 | Adolescents & Adults | Depression | 416 | F | Village-based | No quantitative information of barriers provided by this study | No quantitative information of barriers provided by this study |
| 11 | Yu et al. | 2015 | LY | 18-60 | Adults | Depression & Anxiety | 2,052 | M/F | Rural areas | Participants were asked about their help seeking preference through a question, “When you have mental health problems, what mental healthcare organization would you most likely seek help from?”. Besides, participants were asked reasons for not seeking help. | Percentage of top five reasons for not seeking help (n = 318)  (1) Want to solve it on one’s own (85.2%)  (2) Concern about the cost (43.7%)  (3) Don’t know where to get help (35.5%)  (4) Take too much time or inconvenient (35.2%)  (5) Think treatment is ineffective (32.4%) |
| 12 | Han et al. | 2013 | SJZ | 15-61 | Outpatient & inpatient | Any mental disorder | 342 | M/F | Hospital | 参与者（精神症的患者）被询问，妨碍他们寻求专业心理帮助的原因，问卷列出了19种原因，让受测者对每种因素按“赞同程度”进行评分，在5点李克特（Likert）量表上进行评分。  Participants were asked about barriers to professional mental health help-seeking. There were 19 options provided to participants. Participants can choose their agreement level via the 5-point Likert scale. | 妨碍精神症患者寻求专业心理帮助的原因：   1. 病症外归因（否认自己的问题是心理问题） 2. 治疗负效果（患者对咨询效果的担忧、对心理医生解决问题的能力的怀疑） 3. 咨询业滞后（心理咨询业发展不足） 4. 家庭阻碍（家庭对心理治疗的负面态度） 5. 自我效能（患者认为自己的心理问题并不严重，自己可以自我解决） 6. 面子障碍（羞耻感影响）   *Translation*  Barriers to professional mental health help-seeking:   1. External attribution for mental illness (Denying their mental illness); 2. Worry about the efficacy of the mental health treatment and professionals’ ability; 3. The lagging development of mental health services; 4. Family's opposition (Families’ negative attitude toward mental health treatment); 5. Self-efficacy (Participants believed that they were capable of dealing with their own mental problems) 6. Face concerns (the sense of shame) |
| 13 | Mei, Sui, & Zeng | 1998 | BJ & SH | 18-24 | Students | Depression & anxiety | 466 | M/F | University | 1.參與者被詢問一個開放性問題（未尋求精神科諮詢的可能原因？）  1. Participants were asked an open-ended question (the possible reasons for not seeking psychiatric counselling?)  2.求助傾向  要求參與者對於每一個問題領域，指出以下列哪一種方式解決(a)自己解決(b)向他人求助(c)兩種方式都用  2. Help-seeking tendency  Participants were asked to point out which method they would use under each question’s domain (a) solve by oneself (b)ask others for help (c) both | 不向精神科咨詢的原因   1. 能自己解決 (22.3%) 2. 對精神科醫生不信任 (17.8%) 3. 問題不嚴重 (16.7%) 4. 有其他方法 (11.4%) 5. 丟臉、羞恥、精神病「 烙印」 (9.6%) 6. 看病本身有壓力 (8.5%) 7. 病院可怕 (6.1%) 8. 不願表露 (4.0%) 9. 其他 (3.7%)   其他原因包括:避免見精神病人,不習慣診所的環境,不瞭解精神病咨詢是甚麼,麻煩的程序,位置不便利等等,也阻礙了受測者尋求精神科的咨詢。  *Translation*  Percentage of reasons for not seeking psychiatric care (%).   1. Solve the problems by themselves (22.3%) 2. Distrust of psychiatrists (17.8%) 3. The problem is not serious (16.7) 4. Use other methods (11.4%) 5. Lose face, shame, stigma of mental illness (9.6%) 6. Seeing a doctor itself is stressful (8.5%) 7. Hospital is terrible (6.1%) 8. Unwilling to disclose (4.0%) 9. Other (3.7%)   Other reasons include: avoid seeing psychiatric patients, not adapted to the clinic’s environment, do not know what psychiatric counselling is, cumbersome procedures, inconvenient location, etc. These also prevented subjects from seeking psychiatric counselling. |
| 14 | Wu, Zhu, Liang, & Xu | 2018 | GZ | NR | Students | NR | 383 | M/F | University | 1.心理求助对象  参与者被询问，在遇到心理困扰的时的心理求助对象。  1. Help-seeking objects  Participants were asked about their help-seeking objects when having psychological disturbance.  2. 对心理求助的看法  参与者被询问：“您是否会认为自己或别人有心理求助的想法是一件令人尴尬的事情”、“您认为当自己或别人遇到心理困扰时，没有选择进行心理求助的原因”。  2. Attitudes towards seeking psychological help  Participants were asked “Do you find it embarrassing to have the thought of seeking psychological help, either you yourself or others?”, “What do you think are the reasons why you yourself or others don’t seek psychological help when having psychological disturbance?” | 没有选择进行心理求助的原因：   1. 认为能自主解决问题（75.2%） 2. 认为问题会随着时间的推移而消失 （64.49%） 3. 认为心理求助如心理咨询效率低，周期长，不够及时，效果难以保证（27.42%） 4. 不愿将个人隐私透露于他人，对保密性持有怀疑态度（49.35%） 5. 介意外界对于自己进行心理求助的看法，甚至担心受到歧视（34.73%） 6. 进行心理求助的经济成本和时间成本太高（30.55%） 7. 不了解心理求助（23.24%）   *Translation*  Reasons for nor seeking mental health help:   1. Think the problem can be solved by themselves (75.2%) 2. The problem will go away itself as time goes on (64.49%) 3. Psychological help like psychological counseling has low efficiency, long duration, is not timely enough and the effect cannot be guaranteed. (27.42%) 4. Unwilling to disclose personal privacy to others, hold skeptical attitude to confidentiality. （49.35%） 5. Mind outside’s attitudes towards seeking psychological help, worry about being discriminated. （34.73%） 6. Seeking psychological help is costly in terms of money and time. （30.55%） 7. Have little knowledge of seeking psychological help （23.24%） |

***Note: Author****=First author;* ***Year****=Published year of study;* ***Loc****=Location of study in China (BJ=Beijing; SH=Shanghai; SZ=Shenzhen; LY=Liuyang in Hunan Province; SJZ=Shijiazhuang, Hebei Province; GY=Guangyuan, Sichuan Province; TW=Taiwan; HK=Hong Kong; GZ=Guangzhou, Guangdong Province),* ***Age****=Age of participants;* ***Population****=Participant group characteristics;* ***MHS****=mental health status, i.e. what kind of mental disorder the sample of participants have, e.g. depression, anxiety, stress, PTSD;* ***N****=Total number of participants;* ***Sex****=Gender of participants,* ***M****=Male,* ***F****=Female;* ***Setting****=Where was the study recruited from?. All quantitative studies used survey methodology;* ***NR****=Not reported.;* ***#1- #11*** *= Publications in English;* ***#12 - #14*** *= Publications in Chinese****; #1, #4, & #10****: only quantitative information was extracted in these three mix-method studies.*
