# Supplementary material for: Barriers to Professional Mental Health Help-Seeking Among Chinese Adults: A Systematic Review
Source: Front Psychiatry. 2020 May 20;11:442. doi: 10.3389/fpsyt.2020.00442 (PMC7251144; doi:10.3389/fpsyt.2020.00442)
Supplement: Supplementary file 3 [file DataSheet_3.doc]

**Appendix 3: Qualitative studies included in the review**

**Appendix Table 3: Included qualitative studies on barriers (n=6**)

| **#** | **Author** | **Year** | **Loc** | **Age** | **Population** | **MHS** | **N** | **Sex** | **Setting** | **Method** | 1. **Barrier themes reported** |
| --- | --- | --- | --- | --- | --- | --- | --- | --- | --- | --- | --- |
| **1** | Ma, Tang, & Wang | 2007 | BJ | M=48.65 | Patients | Depression | 112 | M/F | Hospital | Interview | 1. 对精神疾病认识不足(例如：认为精神疾病是躯体问题，情绪低落和思想问题) Misconceptions of mental illness (e.g. think mental illness is physical problems, feeling blue or thought / ideological problems) 2. 家人的阻碍 (Family's opposition) 3. 选取其他途径就诊, 例如综合医院（非精神科）、中医科 Seek help / treatment from other resources, e.g. general hospital - non-psychiatry, Traditional Chinese Medicine Doctor / Traditional Chinese clinics 4. 精神疾病的症状（抑郁症）较轻；Low severity of mental illness (depression) 5. 年龄大, 学历低, 伴有躯体疾病 Sociodemographic barriers, e.g. old age, low level of education, being with physical illness |
| **2** | Gao, Shen & Xu | 2012 | SX | 19-65 | Patients | Depression | 160 | M/F | Hospital | Interview | 对精神疾病认识不足(例如：认为精神疾病是躯体问题，情绪低落和思想问题) Misconceptions of mental illness / Lack of awareness and understanding of mental illness (e.g. think mental illness is ideological problems, and deal with physical symptom) |
| **3** | Yu, Kowitt, Fisher, & Li | 2017 | JN | 16-64 | Patients in a large psychiatric hospital | Depression | 16 | M/F | Hospital | Interview | 1. Fear of stigma to mental illness (public stigma and self-stigma) 2. Deliberately hide the mental illness / Unwillingness to disclose mental illness (patients / patients’ parents) 3. Fear of burdening / influencing their families 4. Misconceptions of mental illness 5. Seek help / care from other resources (e.g. General hospital or traditional Chinese clinics) |
| **4** | Qiu et al. | 2018 | GY | >16 | Adolescents& Adults | Depression | 416 | F | Village-based | Interview | 1. Difficulties in recognizing the mental illness / Limited knowledge about the mental illness 2. Seek help / care from other resources (e.g. family members, friends) 3. Dependence on self, do not want to seek help 4. Low perceived need toward mental health help 5. 5. Negative attitude toward mental illness |
| **5** | Andrade et al. | 2014 | BJ,  SH, &  SZ | 18-88 | Adults | Anxiety & Depression | 9 | M/F | Urban area | Survey (open-end questions) | 1. Barriers to use mental health treatment  1.1 Low perceived need for treatment   1. The problem went away by itself, and I did not really need help.   1.2 Any structural barriers   1. My health insurance would not cover this type of treatment. 2. I was concerned about how much money it would cost. 3. I was unsure about where to go or who to see. 4. I thought it would take too much time or be inconvenient. 5. I could not get an appointment. 6. I had problems with things like transportation, childcare, or scheduling that would have made it hard to get to treatment   1.3 Any attitudinal barriers   1. I thought the problem would get better by itself 2. I didn’t think treatment would work. 3. I was concerned about what others might think if they found out I was in treatment. 4. I wanted to handle the problem on my own. 5. I was scared about being put into a hospital against my will. 6. I was not satisfied with available services. 7. I received treatment before and it did not work. 8. The problem didn’t bother me very much. |
| **6** | Chen | 2018 | BJ | 18-90 | Residents | Psychological distress | 50 | M/F | Urban area | Interview | Barriers to seeking professional mental health help   1. Normalize the mental health problems 2. Tend to resolve mental problems by themselves 3. Seeking mental health help is not necessary and essential 4. Doubt the helpfulness of mental health treatment 5. Deny their mental health problems 6. Previous treatment is not effective 7. Prefer to talk to friends 8. No accessibility of the professional mental health services  - Don't know where to get mental health help - Never made effort to look for mental health help - Doubt the existence of mental health professionals - Not convenient in the distance and time  1. No affordability of the professional mental health services  - Think the mental health service is expensive - Think the the mental health service is only for the rich / high-end consumption people |

***Note: Author****=First author;* ***Year=****Published year of study;* ***Loc****=Location of study (BJ = Beijing, SX=Shaoxing, Zhejiang Province, JN = Jining, Shandong Province, GY = Guangyuan, Sichuan province, SH = Shanghai, SZ = Shenzhen, Guangdong Province) ;****Population=****Participant group characteristics;* ***MHS****=mental health status, i.e.* *what kind of mental disorder the sample of participants have, e.g. depression, anxiety, stress, PTSD;* ***N****=Total number of participants;* ***Sex=****Gender of participants, , M=Male, F=Female;* ***Setting=****Where was the study recruited from?;* ***Method****=Study methodology used; NR=Not reported. #1-2 Publications in Chinese; #3-6 Publications in English. #4-6: Only qualitative information was extracted from these three mix-method studies*
