# Supplementary material for: Barriers to Professional Mental Health Help-Seeking Among Chinese Adults: A Systematic Review
Source: Front Psychiatry. 2020 May 20;11:442. doi: 10.3389/fpsyt.2020.00442 (PMC7251144; doi:10.3389/fpsyt.2020.00442)
Supplement: Supplementary file 4 [file DataSheet_4.docx]

**Appendix 4: Thematic analysis of qualitative studies and CERQual Assessment of Confidence in the Evidence**

**Appendix Table 4: Barrier themes raised by qualitative studies participants and CERQual assessment result (n=6)**

| **Barrier themes raised by qualitative study** | **The terminology used by the study authors was preserved where possible in the descriptions below.** | **CERQual Assessment of Confidence in the Evidence** | **Explanation of CERQual Assessment** | **Studies Contributing to the Review Finding (authors)** |
| --- | --- | --- | --- | --- |
| **1. Seeking help / care from other resources** | (1) General hospital - non-psychiatry (2) TCM / TCC: Traditional Chinese Medicine Doctor / Traditional Chinese Clinics  (3) Family member  (4) Friend | Moderate confidence | This finding was graded as moderate confidence because of moderate concerns regarding methodological limitations and adequacy of data; minor concerns regarding relevance and coherence. | Ma, Tang, & Wang, 2007 Yu, Kowitt, Fisher, & Li, 2017 Qiu et al., 2018 Chen, 2018 |
| **2. Misconceptions for mental illness** | (1) Physical problems / somatization (including decreasing appetite, chest tightness or chest pain, dizziness and headache, limb weakness, insomnia) (2) Feel blue (3) Thought problem / ideological problem (4) Bad thinking (5) Dwelling on bad thoughts (6) Personal flaws and weakness (7) Tachycardia, and early-morning wakefulness | Moderate confidence | This finding was graded as moderate confidence because of moderate concerns regarding methodological limitations; minor concerns regarding relevance, coherence and adequacy of data. | Ma, Tang, & Wang, 2007 Yu, Kowitt, Fisher, & Li, 2017 Gao, Shen & Xu, 2012 |
| **3. Dependence on self and unwillingness to seek help** | (1) Solve the mental problem by themselves / an desire to handle the problem on one's own. (2) Problem will disappear by itself and do not need help (3) Rely on themselves and do not want others to help | Moderate confidence | This finding was graded as moderate confidence because of moderate concerns regarding methodological limitations; minor concerns regarding relevance, coherence and adequacy of data. | Qiu et al., 2018 Andrade et al., 2014 Chen, 2018 |
| **4. Low perceived need for mental health help** | (1) Low perceived need (2) Did not recognize a need (3) Seeking mental health help is not necessary and essential (4) Deny their mental health problems (5) Normalize the mental health problems | Moderate confidence | This finding was graded as moderate confidence because of moderate concerns regarding methodological limitations; minor concerns regarding relevance and coherence; moderate concerns regarding adequacy of data. | Qiu et al., 2018 Andrade et al., 2014 Chen, 2018 |
| **5. Perceived low severity of mental illness** | (1) Low severity of mental illness (depression) (2) The problems did not bother very much (3) Feel better | Moderate confidence | This finding was graded as moderate confidence because of moderate concerns regarding methodological limitations and adequacy of data; minor concerns regarding relevance and coherence. | Ma, Tang, & Wang, 2007 Andrade et al., 2014 |
| **6. Fear of stigma to mental illness** | (1) Public / Social stigma (2) Self-stigma | Moderate confidence | This finding was graded as moderate confidence because of moderate concerns regarding methodological limitations; minor concerns regarding coherence, relevance, and adequacy of data. | Yu, Kowitt, Fisher, & Li, 2017 Andrade et al., 2014 |
| **7. Negative experiences and attitude toward the treatment** | (1) Perceived ineffectiveness of treatment (2) Negative / bad experience with treatment providers (3) Not satisfied with the available resources  (4) The therapist or counselor left or move away (5) Be treated badly or unfairly (6) Feel out of place (7) Previous treatment is not helpful | Moderate confidence | This finding was graded as moderate confidence because of moderate concerns regarding methodological limitations; minor concerns regarding relevance, coherence; and moderate concerns regarding adequacy of data. | Andrade et al., 2014 Chen, 2018 |
| **8. Lack of affordability** | (1) Concerns about the cost (2) Insurance did not cover (3) Fee is too expensive (4) Only for the rich or high-end consumption people | Moderate confidence | This finding was graded as moderate confidence because of moderate concerns regarding methodological limitations; minor concerns regarding relevance, coherence and adequacy of data. | Andrade et al., 2014 Chen, 2018 |
| **9. Lack of accessibility** | (1) Unsure where to get help (2) Lack of transportation (3) Childcare problem (4) Scheduling problem  (5) Policies difficulties (6) Lack of time (7) Moved (8) Cannot get an appointment (9) Never made effort to look for mental health help (10) Doubt the existence of mental health professionals (11) No time for lining up and register (12) Not convenient in the distance (no professional service provided near either their workplace or home) (13) Doubt the helpfulness of mental health treatment | Moderate confidence | This finding was graded as moderate confidence because of moderate concerns regarding methodological limitations; minor concerns regarding relevance, coherence and adequacy of data. | Andrade et al., 2014 Chen, 2018 |
| **10. Families’ opposition** | (1) Families think that the patients' mental illness is result from the weaknesses of personal will, which will delay the help-seeking behavior. (2) Family wants patients to stop the treatment | Low confidence | This finding was graded as low confidence because of moderate concerns regarding methodological limitations, minor concerns regarding relevance and coherence, and substantial concerns regarding adequacy of data. | Ma, Tang, & Wang, 2007 Andrade et al., 2014 |
| **11. Sociodemographic barriers** | (1) Old age (2) Low education background (3) Being with physical illness  (4) Men - masculinity | Low confidence | This finding was graded as low confidence because of moderate concerns regarding methodological limitations and coherence; minor concerns regarding relevance; substantial concerns regarding adequacy of data. | Ma, Tang, & Wang, 2007  Andrade et al., 2014 |
| **12. Unwillingness to disclose mental illness** | (1) Patients deliberately hide their mental illness (2) Parents of a young patient stopped giving the medication and hide the patients' mental illness | Moderate confidence | This finding was graded as moderate confidence because of minor concerns regarding methodological limitations and coherence and adequacy of data; moderate concerns regarding relevance. | Yu, Kowitt, Fisher, & Li, 2017 |
| **13. Fear of burdening their families** | (1) Feel guilt at being disable to fulfill their deities (2) Do not want to burden their families (3) Fear of the impact on family members | Moderate confidence | This finding was graded as moderate confidence because of minor concerns regarding methodological limitations, coherence and adequacy of data; moderate concerns regarding relevance. | Yu, Kowitt, Fisher, & Li, 2017 |
| **14. Difficulties in recognizing the mental illness** | (1) Difficulties in using the specific psychiatric term in description of mental symptoms (2) Reticent to recognize or describe mental symptoms  (3) Do not know if the patients is mentally ill. | Moderate confidence | This finding was graded as moderate confidence because of minor concerns regarding methodological limitations, coherence and adequacy; moderate concerns regarding relevance. | Qiu et al., 2018 |

***Note****: For three mix-method studies (Andrade, 2014; Chen, 2018; Qiu, 2018), only quantitative information was analysed.*
