# Supplementary material for: Barriers to Professional Mental Health Help-Seeking Among Chinese Adults: A Systematic Review
Source: Front Psychiatry. 2020 May 20;11:442. doi: 10.3389/fpsyt.2020.00442 (PMC7251144; doi:10.3389/fpsyt.2020.00442)
Supplement: Supplementary file 5 [file DataSheet_5.docx]

**Appendix 5: Quality assessment for quantitative studies**

**Appendix Table 6.1. Quality assessment for quantitative studies (Tool: QATOCCSS Checklist, n=31)**

| **#** | **1st Author (Year)** | **Q1** | **Q2** | **Q3** | **Q4** | **Q5** | **Q6** | **Q7** | **Q8** | **Q9** | **Q10** | **Q11** | **Q12** | **Q13** | **Q14** |
| --- | --- | --- | --- | --- | --- | --- | --- | --- | --- | --- | --- | --- | --- | --- | --- |
| 1 | Andrade, L. H. (2014) | Yes | Yes | Yes | Yes | No | Yes | NA | NA | Yes | NA | Yes | NA | NA | Yes |
| 2 | Boey, K. W. (1999) | Yes | Yes | Yes | Yes | No | No | NA | NA | No | NA | No | NA | NA | No |
| 3 | Chen, J. (2012) | Yes | Yes | Yes | Yes | No | No | NA | NA | No | NA | No | NA | NA | Yes |
| 4 | Chen, J. (2018) | Yes | Yes | Yes | Yes | No | Yes | NA | NA | Yes | NA | Yes | NA | NA | No |
| 5 | Chen, J. (2018) | Yes | Yes | Yes | Yes | Yes | Yes | NA | NA | Yes | NA | Yes | NA | NA | Yes |
| 6 | Chen, M. T. (2013) | Yes | Yes | Yes | Yes | No | No | NA | NA | Yes | NA | Yes | NA | NA | No |
| 7 | Chin, W. Y. (2015) | Yes | Yes | Yes | Yes | No | No | NA | NA | Yes | NA | Yes | NA | NA | No |
| 8 | Han, Der-Yan (2015) | Yes | Yes | Yes | Yes | No | No | NA | NA | No | NA | No | NA | NA | No |
| 9 | Han, J. (2018) | Yes | Yes | Yes | Yes | No | Yes | NA | NA | Yes | NA | Yes | NA | NA | No |
| 10 | Qiu, P. (2018) | Yes | Yes | Yes | Yes | No | No | NA | NA | No | NA | No | NA | NA | No |
| 11 | Yu, Y. (2015) | Yes | Yes | Yes | Yes | Yes | No | NA | NA | Yes | NA | Yes | NA | NA | Yes |
| 12 | Han, Y. (2013) | Yes | Yes | No | Yes | No | No | NA | NA | No | NA | No | NA | NA | No |
| 13 | Mei, J. R. (1998) | Yes | Yes | Yes | Yes | No | No | NA | NA | No | NA | No | NA | NA | No |
| 14 | Wu, H. H. (2018) | Yes | Yes | Yes | Yes | No | No | NA | NA | No | NA | No | NA | NA | No |

***Note: Yes*** *= relevant information was included in the study****; No*** *= relevant information was NOT included in the study;* ***NA*** *= this item is not applicable for this study.* ***#1- #11*** *= Publications in English;* ***#12 - #14*** *= Publications in Chinese****; #1, #4, & #10****: only quantitative information was assessed in these three mix-method studies.*

| **#** | **1st Author (Year)** | **Quality Rating (Good, Fair, or Poor)** | **Total Score** | **Q1** | **Q2** | **Q3** | **Q4** | **Q5** | **Q6** | **Q9** | **Q11** | **Q14** |
| --- | --- | --- | --- | --- | --- | --- | --- | --- | --- | --- | --- | --- |
| 1 | Andrade, L. H. (2014) | Fair | 13 | 2 | 2 | 2 | 2 | 0 | 0.5 | 2 | 2 | 0.5 |
| 2 | Boey, K. W. (1999) | Poor | 8 | 2 | 2 | 2 | 2 | 0 | 0 | 0 | 0 | 0 |
| 3 | Chen, J. (2012) | Poor | 8.5 | 2 | 2 | 2 | 2 | 0 | 0 | 0 | 0 | 0.5 |
| 4 | Chen, J. (2018) | Fair | 12.5 | 2 | 2 | 2 | 2 | 0 | 0.5 | 2 | 2 | 0 |
| 5 | Chen, J. (2018) | Good | 13.5 | 2 | 2 | 2 | 2 | 0.5 | 0.5 | 2 | 2 | 0.5 |
| 6 | Chen, M. T. (2013) | Fair | 12 | 2 | 2 | 2 | 2 | 0 | 0 | 2 | 2 | 0 |
| 7 | Chin, W. Y. (2015) | Fair | 12.5 | 2 | 2 | 2 | 2 | 0 | 0.5 | 2 | 2 | 0 |
| 8 | Han, Der-Yan (2015) | Poor | 8 | 2 | 2 | 2 | 2 | 0 | 0 | 0 | 0 | 0 |
| 9 | Han, J. (2018) | Fair | 12.5 | 2 | 2 | 2 | 2 | 0 | 0.5 | 2 | 2 | 0 |
| 10 | Qiu, P. (2018) | Poor | 8 | 2 | 2 | 2 | 2 | 0 | 0 | 0 | 0 | 0 |
| 11 | Yu, Y. (2015) | Fair | 13 | 2 | 2 | 2 | 2 | 0.5 | 0 | 2 | 2 | 0.5 |
| 12 | Han, Y. 韩奕(2013) | Poor | 6 | 2 | 2 | 0 | 2 | 0 | 0 | 0 | 0 | 0 |
| 13 | Mei, J. R. (1998) | Poor | 8 | 2 | 2 | 2 | 2 | 0 | 0 | 0 | 0 | 0 |
| 14 | Wu, H. H. (2018) | Poor | 8 | 2 | 2 | 2 | 2 | 0 | 0 | 0 | 0 | 0 |

**Appendix Table 6.2. Quality assessment scoring for quantitative studies (Tool: QATOCCSS Checklist, n=31)**

***Note: #1- #11****=Publications in English;* ***#12 - #14****=Publications in Chinese****; #1, #4, & #10****: only quantitative information was assessed in these three mix-method studies.*

***Scoring:***

***Fatal flaw*** *= Q 1, Q2, Q3, Q4, Q9, Q11 (“Yes “= “2” & “No” =”0”; 2x6 items = 12);* ***Not fatal flaw*** *= Q5, Q6, Q14 (“Yes “= “0.5” & “No” =”0”; 0.5x3 = 1.5);* ***Not applicable (NA) items****: Q7, Q8, Q10, Q12, Q13 (these items were deleted and excluded from scoring);* ***Total score:*** *12 + 1.5 = 13.5*

***Quality Rating:***

***Good:*** *13.5;* ***Fair:*** *11.5 – 13.5;* ***Poor:*** *≤11.5*

**Appendix Table 6.3.** Quality Assessment Tool for Observational Cohort and Cross-Sectional Studies Criteria List (14-item original version)

| Criteria | Y | N | NA |
| --- | --- | --- | --- |
| Q1. Was the research question or objective in this paper clearly stated? |  |  |  |
| Q2. Was the study population clearly specified and defined? |  |  |  |
| Q3. Was the participation rate of eligible persons at least 50%? |  |  |  |
| Q4. Were all the subjects selected or recruited from the same or similar populations (including the same time period)? Were inclusion and exclusion criteria for being in the study pre-specified and applied uniformly to all participants? |  |  |  |
| Q5. Was a sample size justification, power description, or variance and effect estimates provided? |  |  |  |
| Q6. For the analyses in this paper, were the exposure(s) of interest measured prior to the outcome(s) being measured? |  |  |  |
| Q7. Was the timeframe sufficient so that one could reasonably expect to see an association between exposure and outcome if it existed? |  |  |  |
| Q8. For exposures that can vary in amount or level, did the study examine different levels of the exposure as related to the outcome (e.g., categories of exposure, or exposure measured as continuous variable)? |  |  |  |
| Q9. Were the exposure measures (independent variables) clearly defined, valid, reliable, and implemented consistently across all study participants? |  |  |  |
| Q10. Was the exposure(s) assessed more than once over time? |  |  |  |
| Q11. Were the outcome measures (dependent variables) clearly defined, valid, reliable, and implemented consistently across all study participants? |  |  |  |
| Q12. Were the outcome assessors blinded to the exposure status of participants? |  |  |  |
| Q13. Was loss to follow-up after baseline 20% or less? |  |  |  |
| Q14. Were key potential confounding variables measured and adjusted statistically for their impact on the relationship between exposure(s) and outcome(s)? |  |  |  |

*Note:* ***Y****=Yes;* ***N****=No;* ***NA*** *= not applicable*

*Removed items: Q7, Q8, Q10, Q12, Q13 (these items were deleted and excluded from scoring above)*
