# Supplementary material for: Barriers to Professional Mental Health Help-Seeking Among Chinese Adults: A Systematic Review
Source: Front Psychiatry. 2020 May 20;11:442. doi: 10.3389/fpsyt.2020.00442 (PMC7251144; doi:10.3389/fpsyt.2020.00442)
Supplement: Supplementary file 6 [file DataSheet_6.docx]

**Appendix 6: Quality assessment for qualitative studies**

**Appendix Table 6: *Summary of CASP scores of individual studies***

| **#** | **First Author** | **Year published** | **Qualitative research?** | **Clear context?** | **Reflexivity?** | **Clear and appropriate sampling method?** | **Clear and appropriate method of data collection?** | **Clear and appropriate method of analysis?** | **Sufficient evidence?** | **Total Score** | **Methodological limitation (i.e., 1-2 = major; 3-4 = moderate; 5-6 = minor; 7 = none)** |
| --- | --- | --- | --- | --- | --- | --- | --- | --- | --- | --- | --- |
| 1 | Ma, H. M. | 2007 | YES | YES | NO | NO | YES | NO | NO | 3 | Moderate |
| 2 | Yu, S. | 2017 | YES | YES | YES | YES | YES | YES | YES | 7 | None |
| 3 | Qiu, P. | 2018 | YES | YES | NO | YES | YES | YES | YES | 6 | Minor |
| 4 | Andrade, L. H. | 2014 | YES | YES | NO | YES | NO | YES | NO | 4 | Moderate |
| 5 | Gao, S. N. | 2012 | YES | YES | NO | No (not mention selecting method) | YES | NO (only mentioned statistic part) | YES | 4 | Moderate |
| 6 | Juan Chen | 2018 | YES | YES | YES | YES | YES | YES | YES | 7 | None |

***Note:*** *Only quantitative information was assessed in these three mix-method studies (#3, #4, & # 6).*

***Scoring****: Yes = 1; No = 0*
